# Supplementary material for: Age-Related Effects on MSC Immunomodulation, Macrophage Polarization, Apoptosis, and Bone Regeneration Correlate with IL-38 Expression
Source: Int J Mol Sci. 2024 Mar 13;25(6):3252. doi: 10.3390/ijms25063252 (PMC10969909; doi:10.3390/ijms25063252)
Supplement: Supplementary file 1 [file ijms-25-03252-s001.zip › ijms-2800885-supplementary.pptx]

## Slide 1
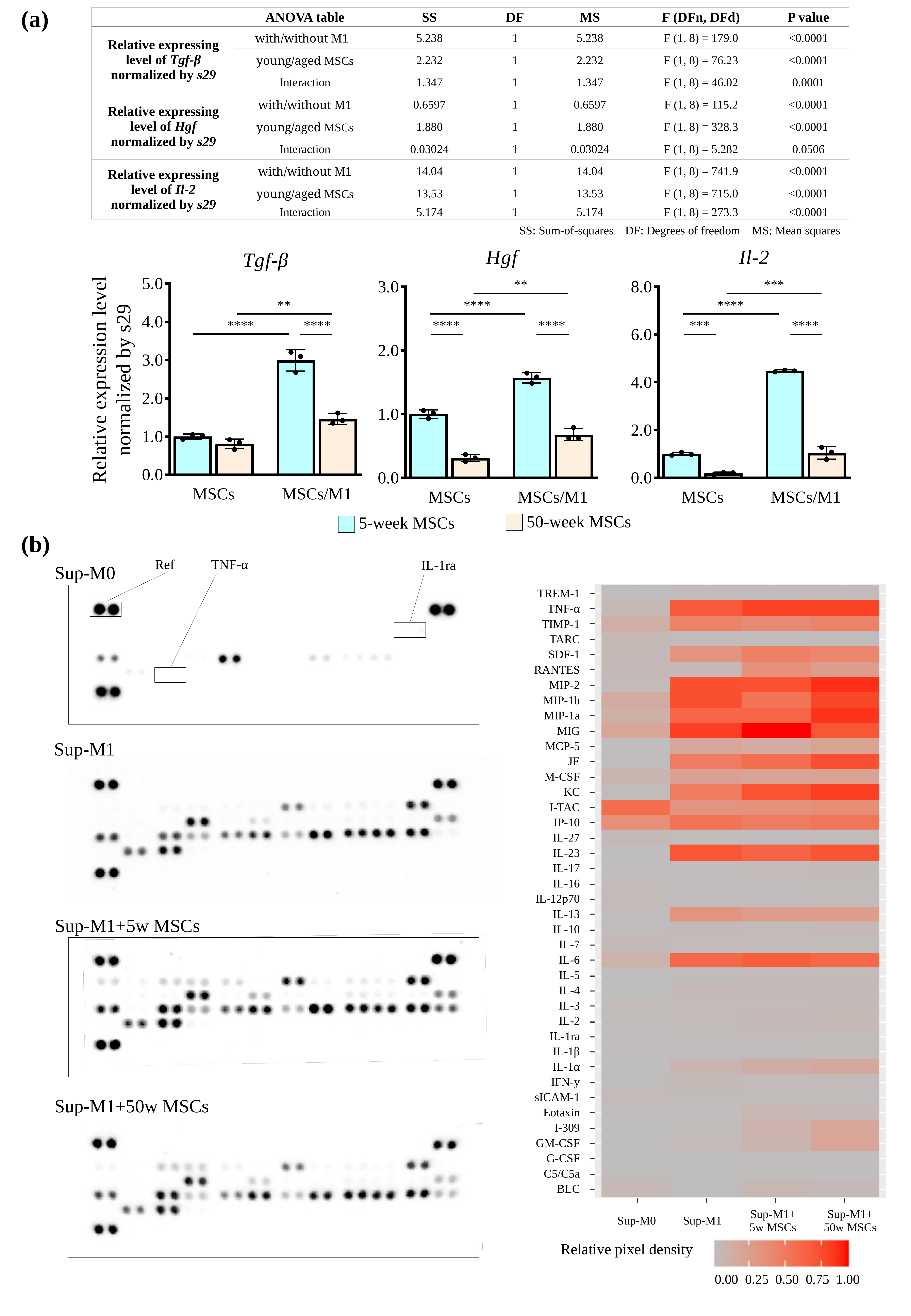

(a)
| | ANOVA table | SS | DF | MS | F (DFn, DFd) | P value |
| --- | --- | --- | --- | --- | --- | --- |
| Relative expressing level of Tgf-β normalized by s29 | with/without M1 | 5.238 | 1 | 5.238 | F (1, 8) = 179.0 | <0.0001 |
| | young/aged MSCs | 2.232 | 1 | 2.232 | F (1, 8) = 76.23 | <0.0001 |
| | Interaction | 1.347 | 1 | 1.347 | F (1, 8) = 46.02 | 0.0001 |
| Relative expressing level of Hgf normalized by s29 | with/without M1 | 0.6597 | 1 | 0.6597 | F (1, 8) = 115.2 | <0.0001 |
| | young/aged MSCs | 1.880 | 1 | 1.880 | F (1, 8) = 328.3 | <0.0001 |
| | Interaction | 0.03024 | 1 | 0.03024 | F (1, 8) = 5.282 | 0.0506 |
| Relative expressing level of Il-2 normalized by s29 | with/without M1 | 14.04 | 1 | 14.04 | F (1, 8) = 741.9 | <0.0001 |
| | young/aged MSCs | 13.53 | 1 | 13.53 | F (1, 8) = 715.0 | <0.0001 |
| | Interaction | 5.174 | 1 | 5.174 | F (1, 8) = 273.3 | <0.0001 |
| SS: Sum-of-squares DF: Degrees of freedom MS: Mean squares | | | | | | |
***
**
**
****
****
****
***
****
****
****
****
50-week MSCs
5-week MSCs
(b)
Ref
TNF-α
IL-1ra
Sup-M0
| TREM-1 |
| --- |
| TNF-α |
| TIMP-1 |
| TARC |
| SDF-1 |
| RANTES |
| MIP-2 |
| MIP-1b |
| MIP-1a |
| MIG |
| MCP-5 |
| JE |
| M-CSF |
| KC |
| I-TAC |
| IP-10 |
| IL-27 |
| IL-23 |
| IL-17 |
| IL-16 |
| IL-12p70 |
| IL-13 |
| IL-10 |
| IL-7 |
| IL-6 |
| IL-5 |
| IL-4 |
| IL-3 |
| IL-2 |
| IL-1ra |
| IL-1β |
| IL-1α |
| IFN-y |
| sICAM-1 |
| Eotaxin |
| I-309 |
| GM-CSF |
| G-CSF |
| C5/C5a |
| BLC |
Sup-M1
Sup-M1+5w MSCs
Sup-M1+50w MSCs
| Sup-M0 | Sup-M1 | Sup-M1+ 5w MSCs | Sup-M1+ 50w MSCs |
| --- | --- | --- | --- |
Relative pixel density
0.00	0.25	0.50	0.75	1.00

## Slide 2
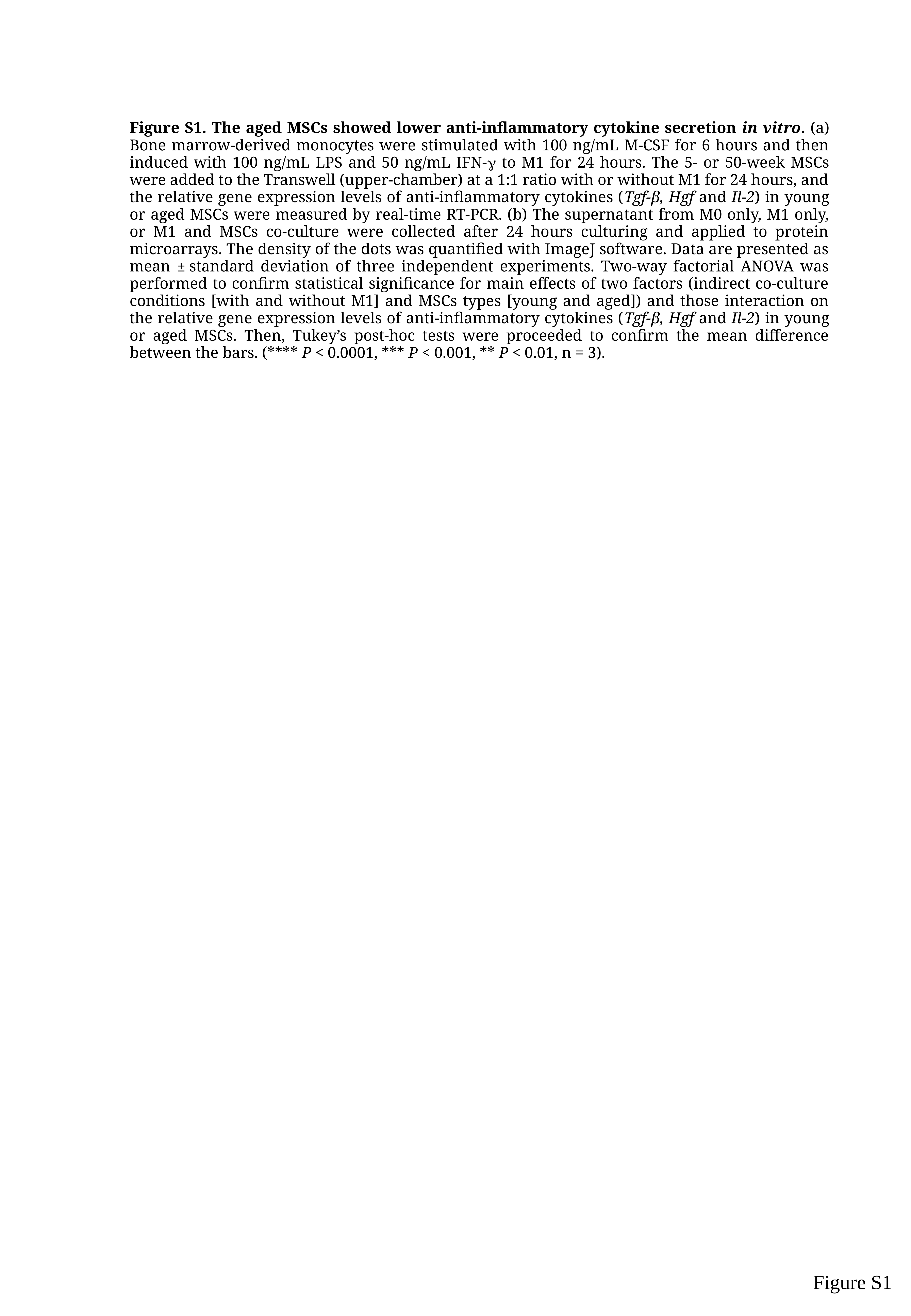

Figure S1. The aged MSCs showed lower anti-inflammatory cytokine secretion in vitro. (a) Bone marrow-derived monocytes were stimulated with 100 ng/mL M-CSF for 6 hours and then induced with 100 ng/mL LPS and 50 ng/mL IFN-γ to M1 for 24 hours. The 5- or 50-week MSCs were added to the Transwell (upper-chamber) at a 1:1 ratio with or without M1 for 24 hours, and the relative gene expression levels of anti-inflammatory cytokines (Tgf-β, Hgf and Il-2) in young or aged MSCs were measured by real-time RT-PCR. (b) The supernatant from M0 only, M1 only, or M1 and MSCs co-culture were collected after 24 hours culturing and applied to protein microarrays. The density of the dots was quantified with ImageJ software. Data are presented as mean ± standard deviation of three independent experiments. Two-way factorial ANOVA was performed to confirm statistical significance for main effects of two factors (indirect co-culture conditions [with and without M1] and MSCs types [young and aged]) and those interaction on the relative gene expression levels of anti-inflammatory cytokines (Tgf-β, Hgf and Il-2) in young or aged MSCs. Then, Tukey’s post-hoc tests were proceeded to confirm the mean difference between the bars. (**** P < 0.0001, *** P < 0.001, ** P < 0.01, n = 3).
Figure S1

## Slide 3
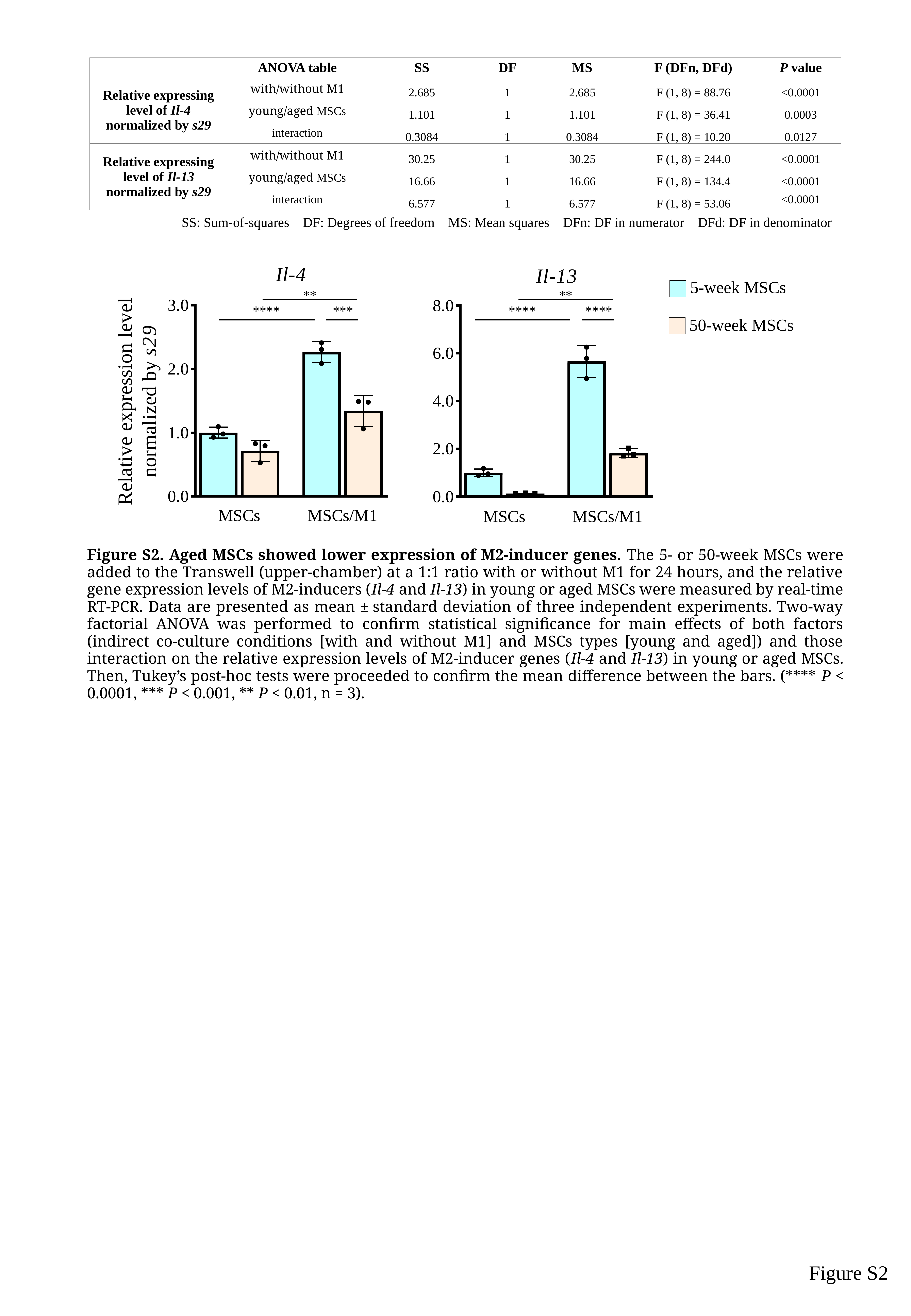

| | ANOVA table | SS | DF | MS | F (DFn, DFd) | P value |
| --- | --- | --- | --- | --- | --- | --- |
| Relative expressing level of Il-4 normalized by s29 | with/without M1 | 2.685 | 1 | 2.685 | F (1, 8) = 88.76 | <0.0001 |
| | young/aged MSCs | 1.101 | 1 | 1.101 | F (1, 8) = 36.41 | 0.0003 |
| | interaction | 0.3084 | 1 | 0.3084 | F (1, 8) = 10.20 | 0.0127 |
| Relative expressing level of Il-13 normalized by s29 | with/without M1 | 30.25 | 1 | 30.25 | F (1, 8) = 244.0 | <0.0001 |
| | young/aged MSCs | 16.66 | 1 | 16.66 | F (1, 8) = 134.4 | <0.0001 |
| | interaction | 6.577 | 1 | 6.577 | F (1, 8) = 53.06 | <0.0001 |
| SS: Sum-of-squares DF: Degrees of freedom MS: Mean squares DFn: DF in numerator DFd: DF in denominator | | | | | | |
5-week MSCs
50-week MSCs
**
**
****
****
***
****
Figure S2. Aged MSCs showed lower expression of M2-inducer genes. The 5- or 50-week MSCs were added to the Transwell (upper-chamber) at a 1:1 ratio with or without M1 for 24 hours, and the relative gene expression levels of M2-inducers (Il-4 and Il-13) in young or aged MSCs were measured by real-time RT-PCR. Data are presented as mean ± standard deviation of three independent experiments. Two-way factorial ANOVA was performed to confirm statistical significance for main effects of both factors (indirect co-culture conditions [with and without M1] and MSCs types [young and aged]) and those interaction on the relative expression levels of M2-inducer genes (Il-4 and Il-13) in young or aged MSCs. Then, Tukey’s post-hoc tests were proceeded to confirm the mean difference between the bars. (**** P < 0.0001, *** P < 0.001, ** P < 0.01, n = 3).
Figure S2

## Slide 4
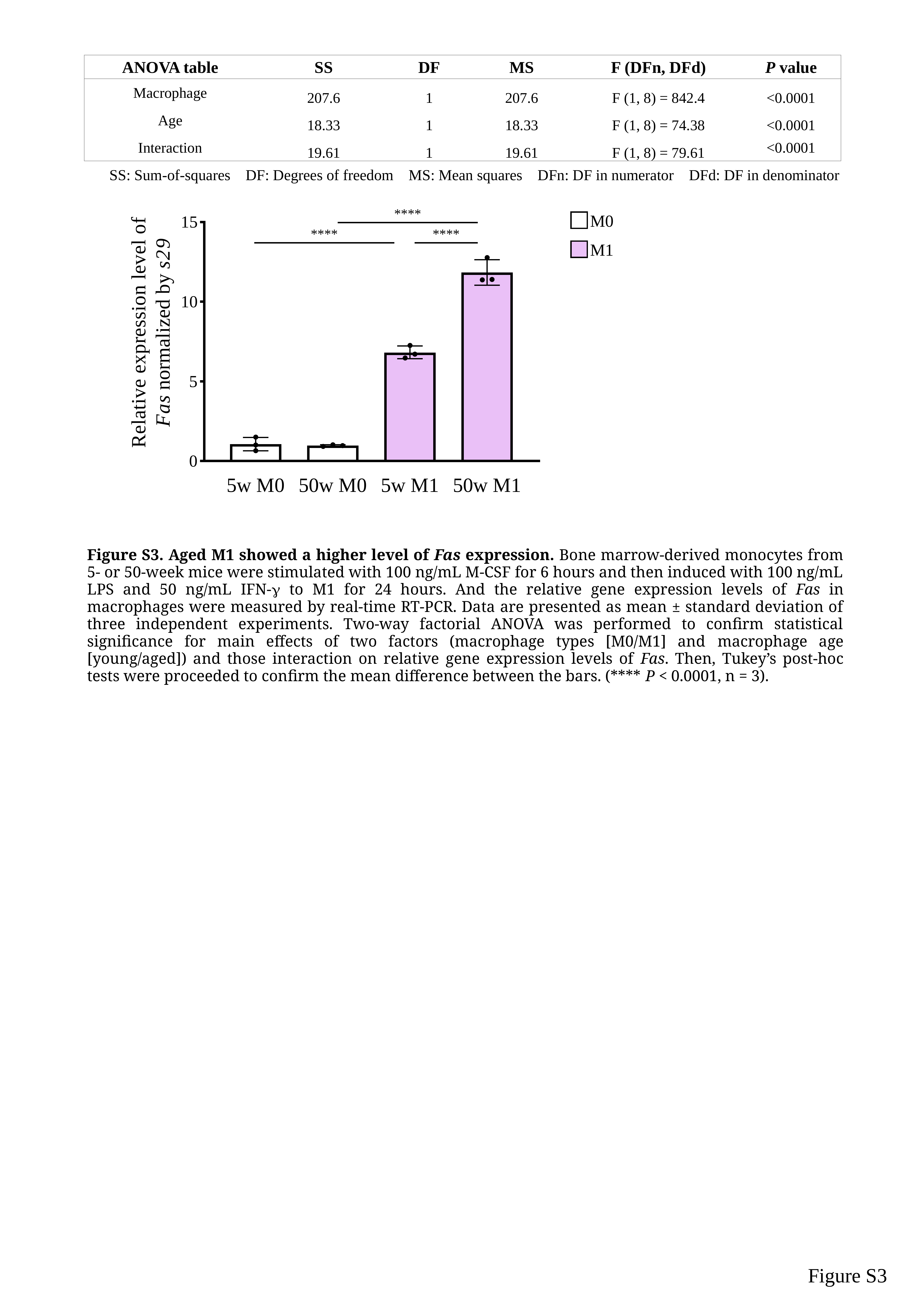

| ANOVA table | SS | DF | MS | F (DFn, DFd) | P value |
| --- | --- | --- | --- | --- | --- |
| Macrophage | 207.6 | 1 | 207.6 | F (1, 8) = 842.4 | <0.0001 |
| Age | 18.33 | 1 | 18.33 | F (1, 8) = 74.38 | <0.0001 |
| Interaction | 19.61 | 1 | 19.61 | F (1, 8) = 79.61 | <0.0001 |
| SS: Sum-of-squares DF: Degrees of freedom MS: Mean squares DFn: DF in numerator DFd: DF in denominator | | | | | |
****
****
****
M0
M1
Figure S3. Aged M1 showed a higher level of Fas expression. Bone marrow-derived monocytes from 5- or 50-week mice were stimulated with 100 ng/mL M-CSF for 6 hours and then induced with 100 ng/mL LPS and 50 ng/mL IFN-γ to M1 for 24 hours. And the relative gene expression levels of Fas in macrophages were measured by real-time RT-PCR. Data are presented as mean ± standard deviation of three independent experiments. Two-way factorial ANOVA was performed to confirm statistical significance for main effects of two factors (macrophage types [M0/M1] and macrophage age [young/aged]) and those interaction on relative gene expression levels of Fas. Then, Tukey’s post-hoc tests were proceeded to confirm the mean difference between the bars. (**** P < 0.0001, n = 3).
Figure S3

## Slide 5
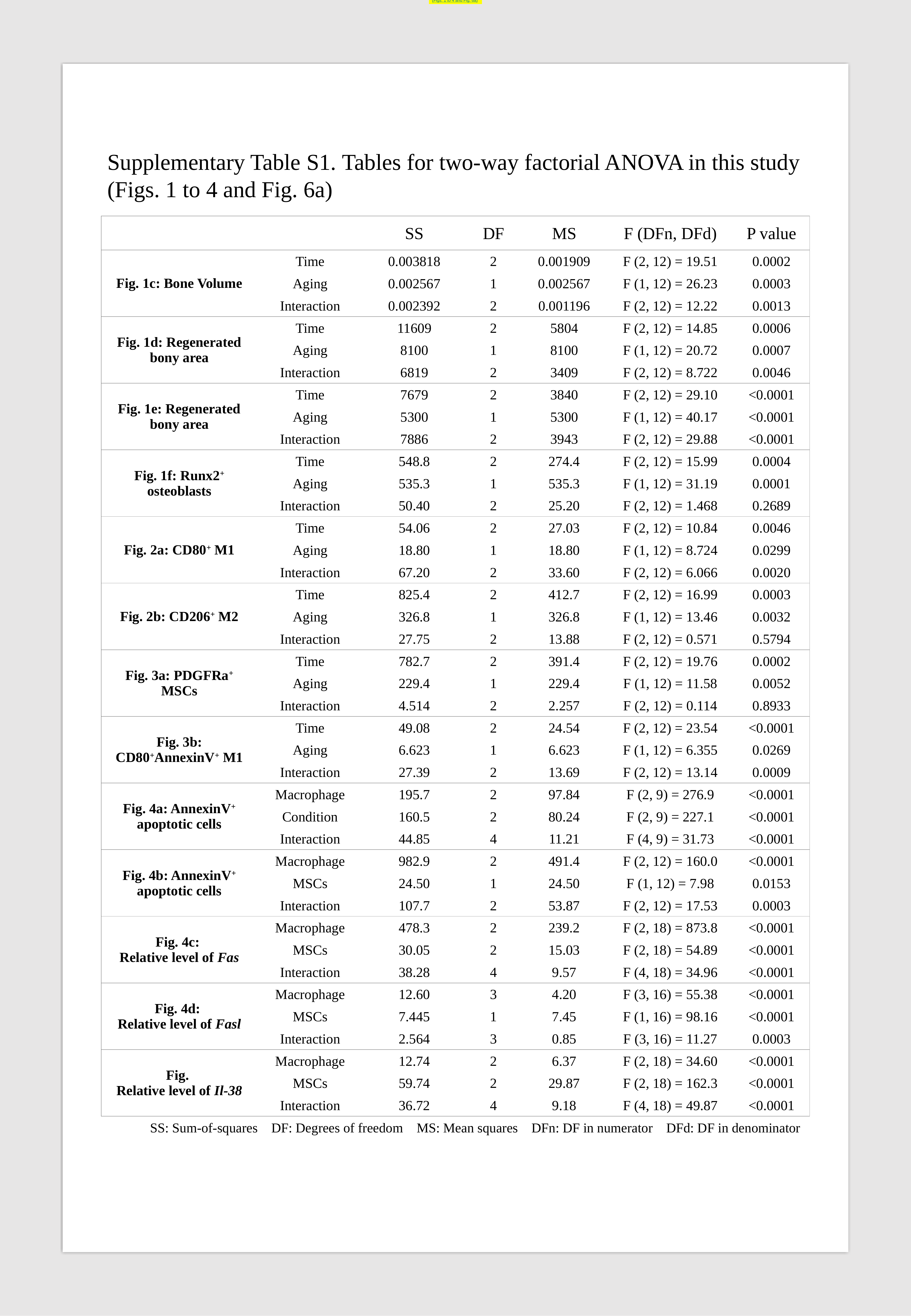

(Figs. 1 to 4 and Fig. 6a)
Supplementary Table S1. Tables for two-way factorial ANOVA in this study
(Figs. 1 to 4 and Fig. 6a)
| | | SS | DF | MS | F (DFn, DFd) | P value |
| --- | --- | --- | --- | --- | --- | --- |
| Fig. 1c: Bone Volume | Time | 0.003818 | 2 | 0.001909 | F (2, 12) = 19.51 | 0.0002 |
| | Aging | 0.002567 | 1 | 0.002567 | F (1, 12) = 26.23 | 0.0003 |
| | Interaction | 0.002392 | 2 | 0.001196 | F (2, 12) = 12.22 | 0.0013 |
| Fig. 1d: Regenerated bony area | Time | 11609 | 2 | 5804 | F (2, 12) = 14.85 | 0.0006 |
| | Aging | 8100 | 1 | 8100 | F (1, 12) = 20.72 | 0.0007 |
| | Interaction | 6819 | 2 | 3409 | F (2, 12) = 8.722 | 0.0046 |
| Fig. 1e: Regenerated bony area | Time | 7679 | 2 | 3840 | F (2, 12) = 29.10 | <0.0001 |
| | Aging | 5300 | 1 | 5300 | F (1, 12) = 40.17 | <0.0001 |
| | Interaction | 7886 | 2 | 3943 | F (2, 12) = 29.88 | <0.0001 |
| Fig. 1f: Runx2+ osteoblasts | Time | 548.8 | 2 | 274.4 | F (2, 12) = 15.99 | 0.0004 |
| | Aging | 535.3 | 1 | 535.3 | F (1, 12) = 31.19 | 0.0001 |
| | Interaction | 50.40 | 2 | 25.20 | F (2, 12) = 1.468 | 0.2689 |
| Fig. 2a: CD80+ M1 | Time | 54.06 | 2 | 27.03 | F (2, 12) = 10.84 | 0.0046 |
| | Aging | 18.80 | 1 | 18.80 | F (1, 12) = 8.724 | 0.0299 |
| | Interaction | 67.20 | 2 | 33.60 | F (2, 12) = 6.066 | 0.0020 |
| Fig. 2b: CD206+ M2 | Time | 825.4 | 2 | 412.7 | F (2, 12) = 16.99 | 0.0003 |
| | Aging | 326.8 | 1 | 326.8 | F (1, 12) = 13.46 | 0.0032 |
| | Interaction | 27.75 | 2 | 13.88 | F (2, 12) = 0.571 | 0.5794 |
| Fig. 3a: PDGFRa+ MSCs | Time | 782.7 | 2 | 391.4 | F (2, 12) = 19.76 | 0.0002 |
| | Aging | 229.4 | 1 | 229.4 | F (1, 12) = 11.58 | 0.0052 |
| | Interaction | 4.514 | 2 | 2.257 | F (2, 12) = 0.114 | 0.8933 |
| Fig. 3b: CD80+AnnexinV+ M1 | Time | 49.08 | 2 | 24.54 | F (2, 12) = 23.54 | <0.0001 |
| | Aging | 6.623 | 1 | 6.623 | F (1, 12) = 6.355 | 0.0269 |
| | Interaction | 27.39 | 2 | 13.69 | F (2, 12) = 13.14 | 0.0009 |
| Fig. 4a: AnnexinV+ apoptotic cells | Macrophage | 195.7 | 2 | 97.84 | F (2, 9) = 276.9 | <0.0001 |
| | Condition | 160.5 | 2 | 80.24 | F (2, 9) = 227.1 | <0.0001 |
| | Interaction | 44.85 | 4 | 11.21 | F (4, 9) = 31.73 | <0.0001 |
| Fig. 4b: AnnexinV+ apoptotic cells | Macrophage | 982.9 | 2 | 491.4 | F (2, 12) = 160.0 | <0.0001 |
| | MSCs | 24.50 | 1 | 24.50 | F (1, 12) = 7.98 | 0.0153 |
| | Interaction | 107.7 | 2 | 53.87 | F (2, 12) = 17.53 | 0.0003 |
| Fig. 4c: Relative level of Fas | Macrophage | 478.3 | 2 | 239.2 | F (2, 18) = 873.8 | <0.0001 |
| | MSCs | 30.05 | 2 | 15.03 | F (2, 18) = 54.89 | <0.0001 |
| | Interaction | 38.28 | 4 | 9.57 | F (4, 18) = 34.96 | <0.0001 |
| Fig. 4d: Relative level of Fasl | Macrophage | 12.60 | 3 | 4.20 | F (3, 16) = 55.38 | <0.0001 |
| | MSCs | 7.445 | 1 | 7.45 | F (1, 16) = 98.16 | <0.0001 |
| | Interaction | 2.564 | 3 | 0.85 | F (3, 16) = 11.27 | 0.0003 |
| Fig. Relative level of Il-38 | Macrophage | 12.74 | 2 | 6.37 | F (2, 18) = 34.60 | <0.0001 |
| | MSCs | 59.74 | 2 | 29.87 | F (2, 18) = 162.3 | <0.0001 |
| | Interaction | 36.72 | 4 | 9.18 | F (4, 18) = 49.87 | <0.0001 |
| SS: Sum-of-squares DF: Degrees of freedom MS: Mean squares DFn: DF in numerator DFd: DF in denominator | | | | | | |

## Slide 6
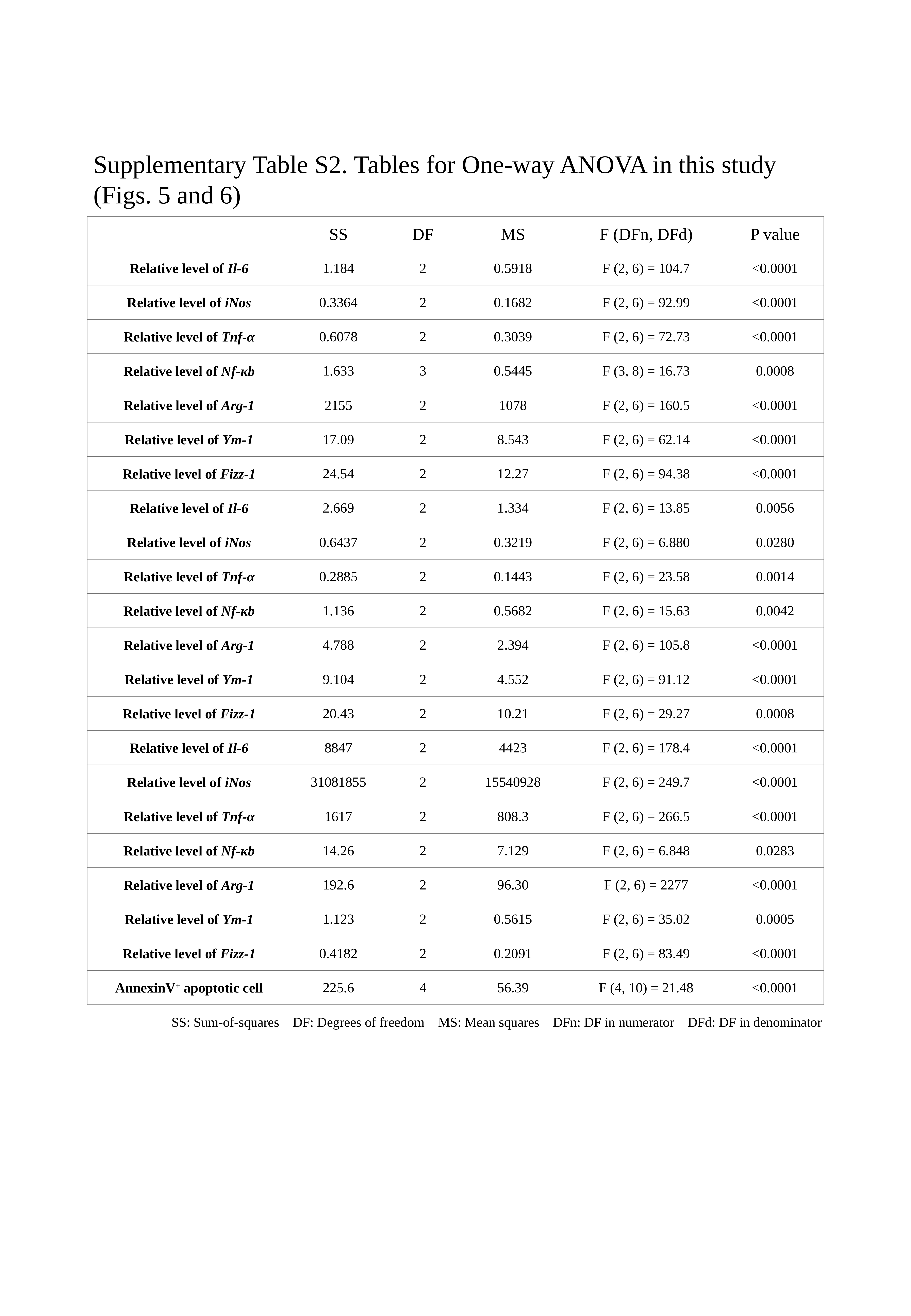

Supplementary Table S2. Tables for One-way ANOVA in this study
(Figs. 5 and 6)
| | SS | DF | MS | F (DFn, DFd) | P value |
| --- | --- | --- | --- | --- | --- |
| Relative level of Il-6 | 1.184 | 2 | 0.5918 | F (2, 6) = 104.7 | <0.0001 |
| Relative level of iNos | 0.3364 | 2 | 0.1682 | F (2, 6) = 92.99 | <0.0001 |
| Relative level of Tnf-α | 0.6078 | 2 | 0.3039 | F (2, 6) = 72.73 | <0.0001 |
| Relative level of Nf-κb | 1.633 | 3 | 0.5445 | F (3, 8) = 16.73 | 0.0008 |
| Relative level of Arg-1 | 2155 | 2 | 1078 | F (2, 6) = 160.5 | <0.0001 |
| Relative level of Ym-1 | 17.09 | 2 | 8.543 | F (2, 6) = 62.14 | <0.0001 |
| Relative level of Fizz-1 | 24.54 | 2 | 12.27 | F (2, 6) = 94.38 | <0.0001 |
| Relative level of Il-6 | 2.669 | 2 | 1.334 | F (2, 6) = 13.85 | 0.0056 |
| Relative level of iNos | 0.6437 | 2 | 0.3219 | F (2, 6) = 6.880 | 0.0280 |
| Relative level of Tnf-α | 0.2885 | 2 | 0.1443 | F (2, 6) = 23.58 | 0.0014 |
| Relative level of Nf-κb | 1.136 | 2 | 0.5682 | F (2, 6) = 15.63 | 0.0042 |
| Relative level of Arg-1 | 4.788 | 2 | 2.394 | F (2, 6) = 105.8 | <0.0001 |
| Relative level of Ym-1 | 9.104 | 2 | 4.552 | F (2, 6) = 91.12 | <0.0001 |
| Relative level of Fizz-1 | 20.43 | 2 | 10.21 | F (2, 6) = 29.27 | 0.0008 |
| Relative level of Il-6 | 8847 | 2 | 4423 | F (2, 6) = 178.4 | <0.0001 |
| Relative level of iNos | 31081855 | 2 | 15540928 | F (2, 6) = 249.7 | <0.0001 |
| Relative level of Tnf-α | 1617 | 2 | 808.3 | F (2, 6) = 266.5 | <0.0001 |
| Relative level of Nf-κb | 14.26 | 2 | 7.129 | F (2, 6) = 6.848 | 0.0283 |
| Relative level of Arg-1 | 192.6 | 2 | 96.30 | F (2, 6) = 2277 | <0.0001 |
| Relative level of Ym-1 | 1.123 | 2 | 0.5615 | F (2, 6) = 35.02 | 0.0005 |
| Relative level of Fizz-1 | 0.4182 | 2 | 0.2091 | F (2, 6) = 83.49 | <0.0001 |
| AnnexinV+ apoptotic cell | 225.6 | 4 | 56.39 | F (4, 10) = 21.48 | <0.0001 |
| SS: Sum-of-squares DF: Degrees of freedom MS: Mean squares DFn: DF in numerator DFd: DF in denominator | | | | | |
